# The significance of the F variant of alpha-1-antitrypsin and unique case report of a PiFF homozygote

**DOI:** 10.1186/1471-2466-14-132

**Published:** 2014-08-07

**Authors:** Nicola Jane Sinden, Firas Koura, Robert Andrew Stockley

**Affiliations:** 1 Centre for Translational Inflammation Research, University of Birmingham, Birmingham, UK; 2 Kentucky Lung Clinic, Hazard, KY, USA; 3 Lung Function and Sleep Department, ADAPT Office (Office 4) Queen Elizabeth Hospital, Birmingham B15 2WB, UK

**Keywords:** Alpha-1-antitrypsin deficiency, Emphysema, Proteinases, Serpin

## Abstract

**Background:**

Inheritance of the F variant of alpha-1-antitrypsin is associated with normal circulating protein levels, but it is believed to be dysfunctional in its ability to inhibit neutrophil elastase and therefore has been implicated as a susceptibility factor for the development of emphysema. In this study, its functional characteristics were determined following the identification of a unique patient with the PiFF phenotype, and the implications as a susceptibility factor for emphysema are considered both in homozygotes and heterozygotes.

**Methods:**

Second order association rate constants were measured for M, Z, S and F variants of alpha-1-antitrypsin with neutrophil elastase and proteinase 3. Clinical characteristics of the PiFF homozygote and six PiFZ heterozygote subjects were studied.

**Results:**

The F variant had a reduced association rate constant with neutrophil elastase (5.60 ± 0.83 × 10^6^ M^-1^ s^-1^) compared to the normal M variant (1.45 ± 0.02 × 10^7^ M^-1^ s^-1^), indicating an increased time to inhibition that was comparable to that of the Z variant (7.34 ± 0.03 × 10^6^ M^-1^ s^-1^). The association rate constant for the F variant and proteinase 3 (1.06 ± 0.22 × 10^6^ M^-1^ s^-1^) was reduced compared to that with neutrophil elastase, but was similar to that of other alpha-1-antitrypsin variants. Of the six PiFZ heterozygotes, five had airflow obstruction and radiological evidence of emphysema. The PiFF homozygote had airflow obstruction but no emphysema. None of the patients had clinical evidence of liver disease.

**Conclusions:**

The F variant may increase susceptibility to elastase-induced lung damage but not emphysema, whereas co-inheritance with the Z deficiency allele may predispose to emphysema despite reasonable plasma concentrations of alpha-1-antitrypsin.

## Background

Alpha-1-antitrypsin deficiency (A1ATD) is a recognised genetic condition that predisposes to the development of early onset basal panacinar emphysema [1]. The pathophysiology is thought to reflect a critically low or absent level of alpha-1-antitrypsin (A1AT) which reduces the ability of the lung to protect itself from neutrophil serine proteinases (NSPs) such as neutrophil elastase (NE) and proteinase 3 (PR3) [2]. The ability of A1AT to protect tissues is both dependent on its concentration and also the speed with which it traps and inactivates these enzymes, its association rate constant (K_ass_). The third NSP inhibited by A1AT, cathepsin G, does not produce emphysema-like lesions in animal models, and has not been included in this study.

The F variant of A1AT was first described in 1965 [3] and results from a single amino acid substitution of arginine at residue 223 by cysteine [4]. It has been reported to have a low K_ass_ for NE [5], and has thus been implicated as a susceptibility factor for emphysema in its own right despite normal serum and hence lung levels. The F variant is relatively uncommon (reported to have an allelic frequency of approximately 0.002 in the Caucasian population [4]) making clinical and epidemiological studies impractical. Even cases of heterozygotes with the Z or other deficiency variants which have both low A1AT levels and reduced K_ass_ for NE are infrequent [6-9], although such patients are often considered for augmentation therapy.

We report a unique case of a patient who is homozygous for the F variant that permitted us to measure the K_ass_ for NE and PR3 with confidence and also review the clinical features both of this patient and of those heterozygous with the Z variant. These data provide insight into whether the F variant represents a risk factor for the development of emphysema, either alone or in combination with the Z variant.

## Methods

### Subject selection

Subjects with A1ATD including PiZZ and PiSS homozygotes and six PiFZ heterozygotes were identified from the U.K. A1ATD national registry (based at Queen Elizabeth Hospital, Birmingham, U.K.) with a confirmed diagnosis by phenotyping and genotyping (Heredilab, Salt Lake City, Utah, USA). One PiFF homozygote was identified from the Kentucky Lung Clinic (Hazard, Kentucky, USA) with a confirmed diagnosis by phenotyping (isoelectric focusing, University of Florida laboratories, Florida, USA). All subjects had demographic data recorded including; medical history, clinical examination, smoking history, quality of life measures (St George’s Respiratory Questionnaire, SGRQ), pulmonary function tests (PFTs), computed tomography (CT) of the thorax, and routine clinical biochemical and haematological parameters. Healthy controls were partners of patients with A1ATD and had a normal PiMM A1AT phenotype. All subjects provided written informed consent for this study, and ethical approval was obtained for this project (South Birmingham Research Ethics Committee LREC 3359).

### Sample collection

Venous blood was collected from all subjects, and serum was obtained using Vacuette^®^ serum tubes (Greiner bio-one, UK) following clot formation. The tubes were centrifuged at 1800 × g for ten minutes at room temperature, and serum was harvested and stored in multiple aliquots at -70°C until analysed, to minimise freeze-thaw cycles.

### Active site titration of enzymes

Pure PR3 (Merck, Feltham, UK) and pure NE (Athens Research and Technology, Athens, Georgia, USA) were active site titrated against pure M variant A1AT (Athens Research and Technology, USA), which had itself been previously titrated against porcine pancreatic elastase (PPE; Sigma-Aldrich, Gillingham, UK). The activity of PPE was determined using Lineweaver–Burk double-reciprocal plot analysis with N-succinyl-Ala-Ala-Ala-p-nitroanilide (SlaaapN; Sigma-Aldrich, UK) as the substrate and published kinetic constants [10].

### Treatment of serum with methylamine to inactivate A2M

In order to measure K_ass_ for serum A1AT with NE or PR3, it was first necessary to inactivate the other major serum inhibitor of NSPs, alpha-2-macroglobulin (A2M). This was achieved by treating the serum samples with 0.1 M methylamine (Fisher Scientific, UK) for 10 minutes at 37°C [11]. The inactivation of A2M was confirmed indirectly by measuring NE and PR3 inhibition by serum samples with and without treatment with methylamine. The substrate used for these experiments was N-Methoxysuccinyl-Ala-Ala-Pro-Val p-nitroanilide (MSaapvN; Sigma-Aldrich, UK) at 0.6 mg/ml in 0.1 M Hepes, pH 7.5, 9.5% dimethyl sulfoxide (DMSO).

### Measurement of K_ass_ for A1AT variants and NE

Pure NE of known activity was diluted to an active concentration of 100nM with buffer (50 mM Hepes, pH 7.4, 150 mM NaCl, 0.05% Igepal CA-630 (v/v)). Methylamine-treated serum samples or pure M or Z variant A1AT were also diluted to an A1AT concentration of 100nM (Z variant A1AT purified from human plasma was a kind gift from the research group of Prof. D. Lomas, University of Cambridge, UK), and these samples were titrated against NE to determine the percentage of active A1AT. Following this initial step, the time-dependent measurements were performed by incubating equimolar amounts of NE and active A1AT (1nM each) in a 1 ml polystyrene cuvette with a 1 cm pathlength at 23°C. Residual NE activity at 1–5 minutes was measured by the addition of 200 μL of MSaapvN and measurement of the change in absorbance (410 nm) over 10 minutes using a spectrophotometer (Jasco V550). At each time point, the percentage of NE inhibition was determined. The K_ass_ was calculated by plotting the inverse of NE activity at each time point versus time. From the linear portion of the slope, the half time (t½) of the reaction was calculated using the formula:

t12=yintercept/slope

K_ass_ was calculated using the equation [12];

Kass=ConcentrationofNE×t12‒1

### Measurement of K_ass_ for A1AT variants and PR3

Pure PR3 of known activity was diluted to an active concentration of 400 nM. Methylamine-treated serum samples or purified M or Z variant A1AT were also diluted to an A1AT concentration of 400 nM and were titrated against PR3 to determine the percentage of active A1AT. Following this initial step, the time-dependent measurements were performed by incubating equimolar amounts of PR3 and active A1AT (1nM each) in a black opaque polypropylene low binding plate (Sigma, UK) at 23°C with a well volume of 150 μL. Residual PR3 activity at 1–5 minutes was measured by adding 3 μL of the PR3 specific Förster resonance energy transfer (FRET) substrate Abz-VAD-norV-ADRQ-EDDnp (Alta Biosciences, UK) at a concentration of 1 mM [13]. The change in fluorescence (excitation 320 nm, emission 420 nm) was measured every 2 minutes for 20 minutes using a Biotek Synergy 2 Multi-Mode Microplate Reader. At each time point, the percentage of PR3 inhibition was determined. The K_ass_ was calculated using the same method as that for NE.

## Results

### Association rate constants for A1AT variants and NSPs

The second order association rate constant (K_ass_) values for different variants of A1AT with NE or PR3 are shown in Table 1. The F variant of A1AT has a reduced K_ass_ value with NE compared to the normal M variant, suggesting that the speed with which it is able to inactivate NE is reduced, and comparable to that observed with the Z variant. The K_ass_ values for A1AT variants and PR3 are reduced compared to those with NE, indicating that NE would be preferentially inhibited in circumstances where the local concentration of A1AT is insufficient to inhibit both NSPs. The results obtained using purified M or Z variant A1AT were comparable to those obtained using methylamine-treated sera (1.47 × 10^7^ M^-1^ s^-1^ for NE and 9.83 × 10^5^ M^-1^ s^-1^ for PR3 with pure M variant A1AT, and 7.39 × 10^6^ M^-1^ s^-1^ for NE and 1.03 × 10^6^ M^-1^ s^-1^ for PR3 with pure Z variant A1AT).

**Table 1:** **K**_**ass **_**values for A1AT variants and NE or PR3**

**A1AT variant**	**Association rate constant with NE**	**Association rate constant with PR3**
	**(Mean ± SEM) at 23°C (M**^**-1**^ **s**^**-1**^**)**	**(Mean ± SEM) at 23°C (M**^**-1**^ **s**^**-1**^**)**
M	(1.45 ± 0.02) × 10^7^	(9.24 ± 0.48) × 10^5^
S	(1.14 ± 0.36) × 10^7^	(9.51 ± 3.00) × 10^5^
Z	(7.34 ± 0.03) × 10^6^	(1.10 ± 0.21) × 10^6^
F	(5.60 ± 0.83) × 10^6^	(1.06 ± 0.22) × 10^6^

### Clinical characteristics of PiFZ heterozygotes

The clinical characteristics of the six PiFZ heterozygote subjects identified from the U.K. A1ATD national registry are summarised in Table 2. All subjects had A1AT concentrations above the putative “protective threshold” of 11 μM and were ex-smokers. Five of the subjects had airflow obstruction on spirometry, as defined by a post-bronchodilator FEV1/FVC ratio <0.7, and these subjects also had radiological evidence of emphysema on high resolution CT (HRCT) scanning, which was panacinar with a lower zone predominance (consistent with the classical distribution and type in PiZZ homozygotes) in two and upper zone predominant in two. The remaining patient had widespread emphysema with no dominant distribution. None of the subjects had biochemical evidence of liver disease, with liver enzymes within the normal range for all subjects.

**Table 2:** Clinical characteristics of six PiFZ heterozygotes identified in the UK

**Clinical parameter**	**Patient 1**	**Patient 2**	**Patient 3**	**Patient 4**	**Patient 5**	**Patient 6**
Age (years)	59	47	70	74	76	53
Sex	M	M	F	M	M	M
A1AT concentration (μM)	18.7	21.6	24.0	11.8	18.3	19.7
Smoking history (pack years)	24	24	23	19	8	40
BMI (kg/m^2^)	23	33	19	29	29	20
FEV1 (% predicted)	29	110	33	99	32	38
FEV1/FVC ratio	0.22	0.77	0.32	0.55	0.31	0.21
KCO (% predicted)	26	100	57	63	87	40
PO_2_ on air (kPa, capillary blood gas)	7.3	8.8	7.8	9.3	7.8	8.8
Average number of exacerbations per year	2	0	2	2	4	1
SGRQ (total score)	63	20	75	42	68	26
CT scan appearance	Upper zone emphysema	Normal	Diffuse emphysema in all zones	Basal emphysema	Basal emphysema & bronchiectasis	Diffuse emphysema, upper zone predominant

### Case report of a PiFF homozygote

A 75 year old caucasian gentleman initially presented to the respiratory clinic in 2004 with a history of increasing exertional dyspnoea over several years and occasional cough productive of green sputum. He was diagnosed with chronic obstructive pulmonary disease (COPD) by spirometry in 2004, but subsequently attended for follow-up only sporadically. He reported having on average 3 pulmonary exacerbations per year. His serial spirometry values are shown in Table 3, with the most recent values showing an obstructive defect, with a pre-bronchodilator FEV1 of 0.75 L (23% predicted), FVC 1.70 L (41% predicted) and FEV1/FVC 44%. His current gas transfer is however within normal limits with DLCO 16.6 mL/mmHg/min (81% predicted) and KCO 2.93 mL/mmHg/min/L (87% predicted). His arterial blood gases (on room air) show pO_2_ 10.1kPa, pCO_2_ 5.2kPa and pH 7.46.

**Table 3:** Serial spirometry from a PiFF homozygote

**Date**	**Pre-bronchodilator FEV1 L (%)**	**Pre-bronchodilator FVC L (%)**	**Pre-bronchodilator FEV1/FVC**	**Post-bronchodilator FEV1 L (%)**	**Post-bronchodilator FVC L (%)**	**Post-bronchodilator FEV1/FVC**
Oct 2004	0.74 (21.0%)	1.65 (37.2%)	44.8	1.05 (29.8%)	2.34 (52.7%)	44.9
July 2005	1.15 (32.6%)	2.14 (48.2%)	53.6	1.24 (35.3%)	2.24 (50.5%)	55.4
May 2007	0.86 (24.9%)	1.93 (44.1%)	44.6	0.97 (28.0%)	2.07 (47.4%)	46.7
Dec 2008	1.04 (33.8%)	2.28 (58.4%)	45.5	Not performed	Not performed	Not performed
Aug 2009	0.96 (28.3%)	2.51 (58.1%)	38.4	1.10 (32.5%)	2.69 (62.3%)	41.0
April 2012	0.74 (22.4%)	1.58 (37.4%)	47.0	Not performed	Not performed	Not performed
Jan 2013	0.75 (23%)	1.70 (41%)	44.1	Not performed	Not performed	Not performed

He has a past medical history of hypertension, hyperlipidaemia, coronary artery disease, pacemaker for second degree heart block and bradycardia, restless legs syndrome and left renal calculus. He has no history of liver disease. His medication consists of salbutamol PRN, fluticasone/salmeterol 500mcg/50mcg bd, montelukast 10 mg od, roflumilast 500 μg od, carbidopa/levodopa 10/100 mg tds, omeprazole 20 mg od, metoprolol 50 mg bd, hydrochlorothiazide 12.5 mg od, rosuvastatin 5 mg od, aspirin 81 mg od, and home oxygen therapy for 18–20 hours per day for 4 years.

He is an ex-smoker having stopped 15 years previously, with a 100 pack year smoking history. There is no history of excessive alcohol intake or illicit drug use. He is retired, but previous occupations include working in school maintenance, as a bus driver, strip coal mining for 4–5 years, and as a carpenter for 20 years. His father had asthma and “bronchitis” and was a lifelong non-smoker. His mother was also a non-smoker and had no respiratory problems. He has no siblings and has 3 children who are healthy and 2 grandchildren, one of whom has asthma but a normal PiM phenotype.

On physical examination, his body mass index (BMI) was 25.8 kg/m^2^. He had an oxygen saturation of 96% on room air, and clear lungs on auscultation with good bilateral air entry. Cardiovascular examination was normal and there was no peripheral oedema, cyanosis or finger clubbing.

**Figure 1:**
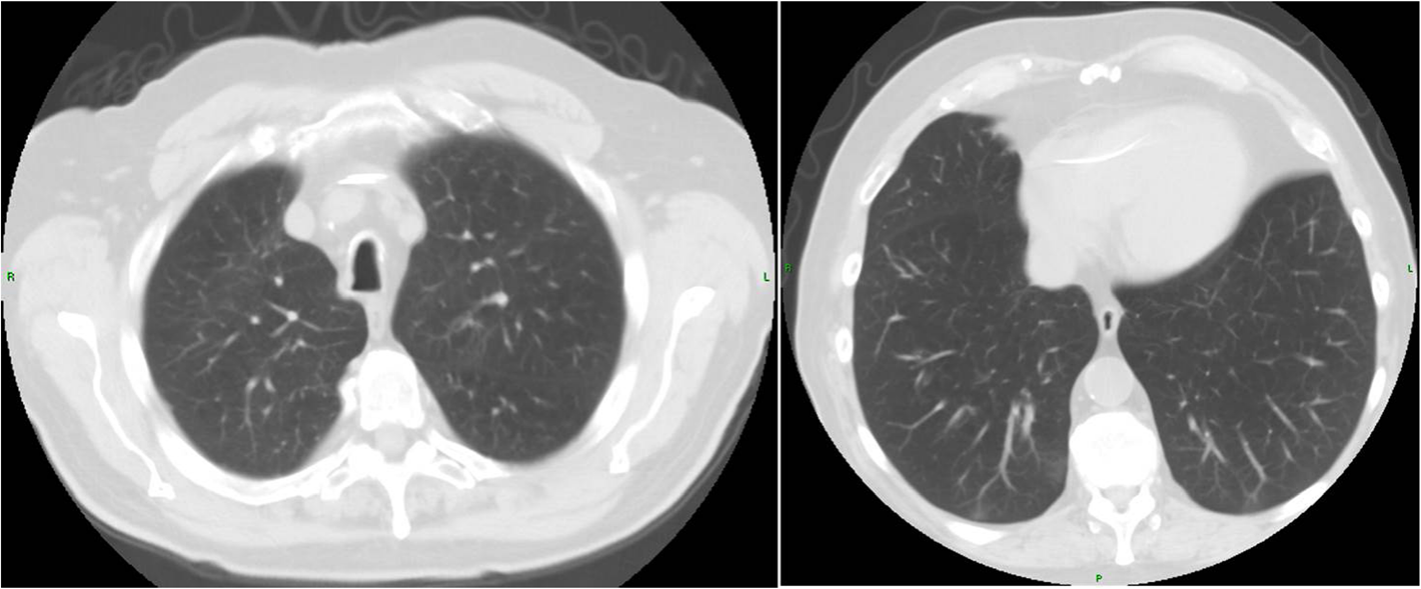
**HRCT thorax of the PiFF homozygote.** There is no radiological evidence of emphysema or bronchiectasis.

His A1AT concentration was 37.7 μM by nephelometry and phenotype PiFF by isoelectric focusing. Routine haematological parameters and hepatic and renal function tests were within normal limits. His SGRQ total score was 73.A HRCT scan of the thorax performed in 2013 showed fibrosis and pleural thickening at the apices and bronchial wall thickening around the hilum, but no radiological evidence of emphysema or bronchiectasis (as shown in Figure 1). An echocardiogram showed a left ventricular ejection fraction of 55-60%, and normal right ventricular function with no evidence of pulmonary hypertension.

## Discussion

We have used the unique identification of a patient with the PiFF phenotype of A1ATD to ascertain the function of F variant A1AT, and consider whether its inheritance represents a susceptibility factor for developing emphysema, in either the homozygous or heterozygous state. The F variant of A1AT has generally been thought not to be clinically important because of its near normal circulating levels [14]. However, previous reports have suggested that the F variant has abnormal function, and may therefore predispose to the development of lung or liver disease (typical of the Z variant) when co-inherited with another deficiency gene as in the PiFZ heterozygote. In 1974, Brand et al. [15] reported a case of liver cirrhosis in a PiFZ heterozygote, although the subject had no evidence of emphysema. Subsequently in 1981, Cockcroft et al. [6] reported 3 PiFZ siblings who all had moderate to severe COPD, two of whom had radiological evidence of emphysema. Their six living siblings (3 PiFM, 2 PiMM and 1 PiMZ) had milder abnormalities in pulmonary function despite similar smoking histories and exposure to grain dust suggesting that the FZ combination was a susceptibility factor. In 1982, Billingsley and Cox [14] reported that PiFM serum showed reduced specific activity with elastase (PPE) compared to the normal PiMM variant. Subsequently, Beckman et al. [7] in 1984 reported that in 13 individuals heterozygous for the F variant of A1AT, respiratory symptoms were present in seven out of twelve PiFM heterozygotes, of whom five had abnormal PFTs, and one PiFZ heterozygote who had bronchitic symptoms and mild airflow obstruction on spirometry. They hypothesized that an increased risk for pulmonary disease may be due to dysfunction of the F protein, independent of the A1AT concentration. A case report by Kelly et al. [8] described a 56 year old PiFZ lady with a 10 pack year smoking history and minimal alcohol intake who presented with advanced emphysema, cor pulmonale and abnormalities on liver biopsy consistent with A1ATD. More recently, a 64 year old male with the PiFnull phenotype has been reported [9]. This patient had a 10 pack year smoking history and moderate COPD, with no biochemical evidence of liver disease. This patient was commenced on A1AT augmentation therapy with no further decline in lung function or symptoms reported.

The cases described in the present study demonstrate that five of the six unrelated PiFZ heterozygotes identified from the U.K. A1ATD national registry had COPD with radiological evidence of emphysema, although none had biochemical evidence of liver disease. The A1AT levels were within the “normal” range and consistent with those of PiMZ heterozygotes [16] although only two had the classical basal distribution of emphysema typical of PiZZ homozygotes. Whether this level of A1AT predisposes to the development of COPD however, remains uncertain. The patients were referred to the U.K. registry because of the identification of the PiFZ phenotype and may therefore reflect an acquisition bias. The PiMZ heterozygous state has long been studied as a potential risk factor and a meta-analysis does suggest a slight increase in susceptibility [17], with the heterozygous state being associated with more hospitalisations [18] and a more severe form of COPD [19,20]. An additional risk might be conferred by the proposed reduction in K_ass_ for NE by the F protein. The PiFF homozygote reported here enabled us to confirm the reduction in K_ass_ with confidence, but despite this and a significant smoking history, the patient did not have radiological evidence of emphysema or impaired transfer factor, although he did have significant physiological impairment on spirometry. His A1AT concentration was within the normal range of PiMM homozygotes [16]. The absence of emphysema despite a significant smoking history suggests that homozygosity for the F variant is not a significant risk factor for the development of emphysema. It remains possible, however, that co-inheritance of the F variant of A1AT with another deficiency allele does increase the risk of developing emphysema, although the distribution in the PiFZ patients reported here is more consistent with usual COPD and PiSZ heterozygotes [21] than PiZZ homozygotes, suggesting a different mechanism. In PiMZ heterozygotes, the increased susceptibility to developing COPD is accentuated in ever-smokers [20]. It could therefore be postulated that cigarette smoking in PiFZ heterozygotes could lead to a more severe form of COPD than in PiMZ heterozygotes due to the reduced K_ass_ for NE by the F protein. Whether A1AT augmentation therapy should be considered for PiFZ heterozygotes remains unknown since the A1AT concentration is low and both the F and Z variants have reduced K_ass_ for NE.

Our cases have not demonstrated any association between the F variant of A1AT and liver disease, although case reports have been described elsewhere in PiFZ heterozygotes [8,15]. The loop sheet polymers formed by the Z protein are retained within the rough endoplasmic reticulum of hepatocytes forming periodic acid Schiff (PAS) positive hepatic inclusions [22], and this accumulation can lead to liver disease including hepatitis, cirrhosis and hepatocellular carcinoma [23] in PiZZ homozygotes. Co-inheritance of the Z variant as a heterozygote will still form polymers and even heteropolymers [24], and although the susceptibility of the F variant to polymerise has not been reported in the literature to date, it remains a possible mechanism whereby some PiFZ heterozygotes may be at increased risk of developing liver disease.

In future studies, it would be ideal to use purified F variant A1AT to measure K_ass_ values with the NSPs. However, the results presented here using purified M or Z variant A1AT were comparable to those obtained using methylamine-treated sera, supporting the use of this method for the F variant of A1AT. In addition, our results are consistent with those reported elsewhere by Cook et al. [5], who used partially purified F variant A1AT.

A potential mechanism for the impaired function of the F variant may relate to the position of the mutation (arginine replaced by cysteine at position 223). Examination of the structure of A1AT shows that the mutation is in the C-sheet underlying the reactive centre loop (RCL). With the normal M variant, an arginine at position 223 forms an electrostatic interaction with the side chain of glutamate 354 in the RCL [25]. However, with the F variant, the cysteine at position 223 cannot make this electrostatic interaction and the consequence is likely to be a disruption of the RCL architecture which alters the conformation of the P1-P1’ region (the bond cleaved by target NSPs) and may explain the reduced K_ass_ with NE observed here.

The data presented here also confirms that F A1AT has a similar K_ass_ for PR3 to that of the normal M variant, suggesting a less significant effect of mutations in A1AT on its ability to inhibit PR3. Therefore, inhibition of PR3 activity is likely to be more dependent on local A1AT concentrations than K_ass_. Previous studies have suggested that PR3 activity is inhibited only when NE has been totally inhibited [26], and therefore in situations where there is insufficient A1AT to inhibit both NSPs, NE will be preferentially inhibited. PR3 can replicate the pathological features of COPD associated classically with NE and should be considered when evaluating the proteinase/anti-proteinase imbalance observed in A1ATD [27], as its activity is likely to persist longer in the lung in the presence of neutrophilic inflammation. Whether the reduced A1AT concentration in PiFZ heterozygotes and the reduced K_ass_ for NE alters this relationship remains to be determined.

## Conclusion

We have demonstrated that the F variant of A1AT has a reduced functional ability to inhibit NE (but not PR3) compared to the normal M variant. This may be of clinical significance when the F variant is co-inherited with the Z variant or another deficiency variant, although its significance is less certain in the homozygous state when concentrations of A1AT are normal.

## Abbreviations

A1AT: Alpha-1-antitrypsin; A1ATD: Alpha-1-antitrypsin deficiency; A2M: Alpha-2-Macroglobulin; BMI: Body mass index; COPD: Chronic obstructive pulmonary disease; CT: Computed tomography; DLCO: Carbon monoxide diffusing capacity; DMSO: Dimethyl sulfoxide; FEV1: Forced expiratory volume in one second; FRET: Förster resonance energy transfer; FVC: Forced vital capacity; HRCT: High resolution computed tomography; K_ass_: Second order association rate constant; KCO: Carbon monoxide transfer coefficient; MSaapvN: N-Methoxysuccinyl-Ala-Ala-Pro-Val p-nitroanilide; NE: Neutrophil elastase; NSP: Neutrophil serine proteinase; PFTs: Pulmonary function tests; PPE: Porcine pancreatic elastase; PR3: Proteinase 3; RCL: Reactive centre loop; SEM: Standard error of the mean; SGRQ: St George’s Respiratory Questionnaire; SlaaapN: N-succinyl-Ala-Ala-Ala-p-nitroanilide.

## Competing interests

The A1ATD U.K. national registry was funded by an unrestricted educational grant from Grifols Therapeutics. RS has sat on advisory boards for CSL, Kamada and Grifols; has received lecture fees from CSL and Grifols, and has received unrestricted research grants from CSL and Grifols. FK has sat on advisory boards for Grifols, Baxter and CSL, and received speaker's fees from Grifols and Baxter.

## Authors’ contributions

NS performed the laboratory experiments, gathered the data on the PiFZ heterozygotes and wrote the article. FK gathered all data from the PiFF homozygote and collected the necessary samples for the laboratory experiments. RS runs the U.K. A1ATD national registry, designed the concept of the study and revised the article. All authors read and approved the final manuscript.

## Pre-publication history

The pre-publication history for this paper can be accessed here:

http://www.biomedcentral.com/1471-2466/14/132/prepub
